# Impact of aberrant cerebral perfusion on resting-state functional MRI: A preliminary investigation of Moyamoya disease

**DOI:** 10.1371/journal.pone.0176461

**Published:** 2017-04-25

**Authors:** Yituo Wang, Lubin Wang, Penggang Qiao, Fugeng Sheng, Cong Han, Enmao Ye, Yu Lei, Feng Yan, Shanshan Chen, Yuyang Zhu, Guiyun Mi, Gongjie Li, Zheng Yang

**Affiliations:** 1 Cognitive and Mental Health Research Center, Beijing Institute of Basic Medical Sciences, Beijing, People’s Republic of China; 2 Department of Radiology, Affiliated Hospital of the Academy of Military Medical Sciences, Beijing, People’s Republic of China; 3 Department of Neurosurgery, Affiliated Hospital of the Academy of Military Medical Sciences, Beijing, People’s Republic of China; Banner Alzheimer's Institute, UNITED STATES

## Abstract

The impact of chronic cerebral hypoperfusion on resting-state blood oxygen level-dependent signal fluctuations remains unknown. We aimed to determine whether chronic ischemia induces changes in amplitude of low-frequency fluctuations (ALFF) and to investigate the correlation between ALFF and perfusion-weighted magnetic resonance imaging (PWI) parameters in patients with moyamoya disease (MMD). Thirty patients with pre- and postoperative resting-state functional magnetic resonance imaging and PWI were included, and thirty normal controls underwent resting-state functional magnetic resonance imaging. A decrease in preoperative frontal lobe ALFF was observed in patients with MMD. Postoperative frontal lobe ALFF showed moderate improvement but still remained lower than those in normal controls. The values of mean transit time and time-to-peak, but not cerebral blood volume and cerebral blood flow, correlated significantly with frontal lobe ALFF. Moreover, there were significant negative correlations between changes in frontal lobe PWI parameters and changes in frontal lobe ALFF on both operated side and contralateral side after the unilateral revascularization surgery. Our results demonstrate that reduced ALFF are closely related to the abnormal PWI parameters and vary with the alteration of cerebral perfusion in patients with MMD.

## Introduction

Resting-state functional magnetic resonance imaging (rs-fMRI) is typically used to investigate the functional organization of the brain.[1] However, spontaneous blood oxygen level-dependent (BOLD) signal fluctuations can also be influenced by several physiological factors, such as cerebral perfusion status. The strongest spontaneous BOLD signal fluctuations have been observed in the posterior cingulate cortex, precuneus, and medial prefrontal cortex,[2] where the cerebral blood flow (CBF) and cerebral metabolic rate of oxygen (CMRO_2_) were higher than in other brain regions.[3] Furthermore, temporal-delayed resting-state BOLD signal fluctuations have been shown to correlate with mean transit time (MTT) in patients with acute stroke[4] and with time-to-peak (TTP) in patients with moyamoya disease (MMD), a progressive cerebrovascular condition characterized by blocked arteries at the base of the brain.[5] Therefore, besides neural activity, spontaneous BOLD signal fluctuations may contain information on brain perfusion and cerebral vascular reactivity (CVR).

Spontaneous low-frequency fluctuations (0.01–0.1 Hz) in BOLD signals have been considered to reflect neural synchronization and cortical excitability.[6,7] Recently, the amplitude of low-frequency fluctuations (ALFF) in rs-fMRI has increasingly been used to detect the spatial pattern of baseline brain activity in both healthy participants[8,9] and patients with attention deficit hyperactivity disorder, schizophrenia, or Alzheimer’s disease.[10,11,12] However, the relationship between ALFF and brain perfusion has not been elucidated. After observing that ALFF may reflect cerebral physiological states of the brain,[8] we hypothesized that ALFF would be affected by the abnormal cerebral perfusion in cerebrovascular diseases.

In this study, we compared ALFF between normal controls and patients with MMD. This rare cerebrovascular disease is characterized by progressive stenosis and occlusion of the terminal internal carotid arteries and their main branches, with collateral formation.[13,14] As such, these patients may have reduced CBF[15,16,17], increased cerebral blood volume (CBV)[18], MTT[19] and TTP.[20] We investigated whether ALFF were related to these perfusion-weighted magnetic resonance imaging (PWI) parameters and whether changes in ALFF correspond with those in PWI parameters after revascularization surgery.

## Materials and methods

### Participants

The Research Ethics Committee of the Affiliated Hospital of the Academy of Military Medical Sciences approved this retrospective study; the requirement for informed consent was waived.

From June 2014 to July 2015, 157 consecutive adults with MMD underwent revascularization surgery at our hospital. Of these, 30 patients (14 male, 16 female; mean age, 35.6 years; range, 19–59 years) who met the following criteria were recruited: (1) Patients who were more than 18-years-old and with a diagnosis confirmed by digital subtraction angiography. (2) Patients who had undergone pre- and postoperative rs-fMRI and PWI (43 patients were excluded). (3) Patients who only underwent the unilateral revascularization surgeries and the follow-up rs-fMRI and PWI scanning were more than 3 months after the unilateral procedures. (4) Patients in whom the cerebrovascular accident (CVA) scores (the ischemic or hemorrhagic CVA lesions were divided into the following categories; point 0 = none, point 1 = small CVA lesion (longest diameter, <1 cm), point 2 = medium CVA lesion (longest diameter, 1–3 cm), point 3 = large CVA lesion (longest diameter, >3 cm))[21] in the bilateral frontal lobes were ≤ point 1 by using fluid attenuated inversion recovery (FLAIR) imaging with the purpose of minimizing the potential biases of ischemic and/or hemorrhagic lesions on BOLD signals (84 patients were excluded).

Thirty young, healthy, right-handed university students (male: 16; female: 14; age range: 21–25 years; mean: 22.9 years) were recruited as normal controls (NC) in this study. All subjects reported the absence of a history of neurological, psychiatric, and cerebrovascular disorders, and of alcohol/drug abuse, and reported no current use of any psychoactive drugs or corticosteroids. None of them had experienced severe head trauma. All subjects gave written informed consent to participate in this study, which was approved by the Research Ethics Committee of Affiliated Hospital of the Academy of Military Medical Sciences.

### MRI data acquisition

#### Resting-state fMRI

Rs-fMRI imaging data were collected using a 3.0-T Skyra MR scanner (Siemens, Erlangen, Germany) equipped with high-speed gradients and a standard birdcage head-coil. All participants were restrained by foam pads in order to minimize head motion. After a 2D-localizer scan, rs-fMRI images were obtained with an echo-planar imaging (EPI) sequence during 6 min and 23 s scanning (150 functional volumes in total), 43 contiguous slices with a slice thickness of 4 mm; repetition time (TR), 2500 ms; echo time (TE), 30 ms; flip angle (FA), 90°; field of view (FOV), 210 × 210 mm^2^; data matrix, 64 × 64 were obtained. During the rs-fMRI scanning, all participants were instructed to keep their eyes closed, stay awake, and not to think about anything in particular during the entire session.

#### Perfusion MR imaging

PWI data were obtained using a spin-echo EPI (SE-EPI) sequence with the following parameters: TR, 1870 ms; TE, 30 ms; slice thickness, 4 mm; FOV, 220 × 220 mm^2^; data matrix, 128 × 128. A series of images (60 sections, 24 images per section) was obtained before and after the injection of gadopentetate dimeglumine (0.1 mmol per kilogram of body weight, at a rate of 4 ml per s, manually).

#### Fluid attenuated inversion recovery imaging

FLAIR data were performed using a fast inversion recovery sequence with the following parameters: TR, 9000 ms; TE, 85 ms; FA, 150°; slice thickness, 5 mm; number of slices, 20; FOV, 220 × 180–220 mm^2^.

### MRI data analysis

#### Resting-state fMRI data analysis

Rs-fMRI data were preprocessed using a MATLAB toolbox, the Data Processing Assistant for Resting-State (DPARSF) V2.1 Basic Edition,[22] which was based on statistical parametric mapping software functions (SPM8; http://www.fil.ion.ucl.ac.uk/spm/software/spm8) and REST software (http://restfmri.net/forum/index.php). For each participant, the first five time-points were discarded to avoid transient signal changes before magnetization reached a steady-state and to allow subjects to become accustomed to the fMRI scanning noise. The rs-fMRI data were corrected for the acquisition delay among the slices. It has been previously been showed in various studies that spurious correlations appear due to head motion artifact.[23] Therefore, we preferred the Friston 24-parameter model (including 6 head motion parameters of the current time point and the preceding time point, and the 12 corresponding square items)[24] for motion correction, because higher-order regression models may perform better at the individual-subject level.[25] Furthermore, a threshold for head motion during the present study was established at 3 mm and 3 degrees. Following motion correction, all data were spatially normalized to the Montreal Neurological Institute (MNI) template and resampled to 3 × 3 × 3 mm^3^. Then, the processed images were spatially smoothed with a 6-mm full-width half-maximum Gaussian kernel, followed by linear detrending to remove any residual drift. Twenty-seven nuisance signals were removed from the time series of each voxel via linear regression, including the global signal, white matter signal, cerebrospinal fluid signal and twenty-four head motion parameters. This regression procedure was utilized to reduce spurious variance unlikely to reflect neural activity.

**Fig 1:**
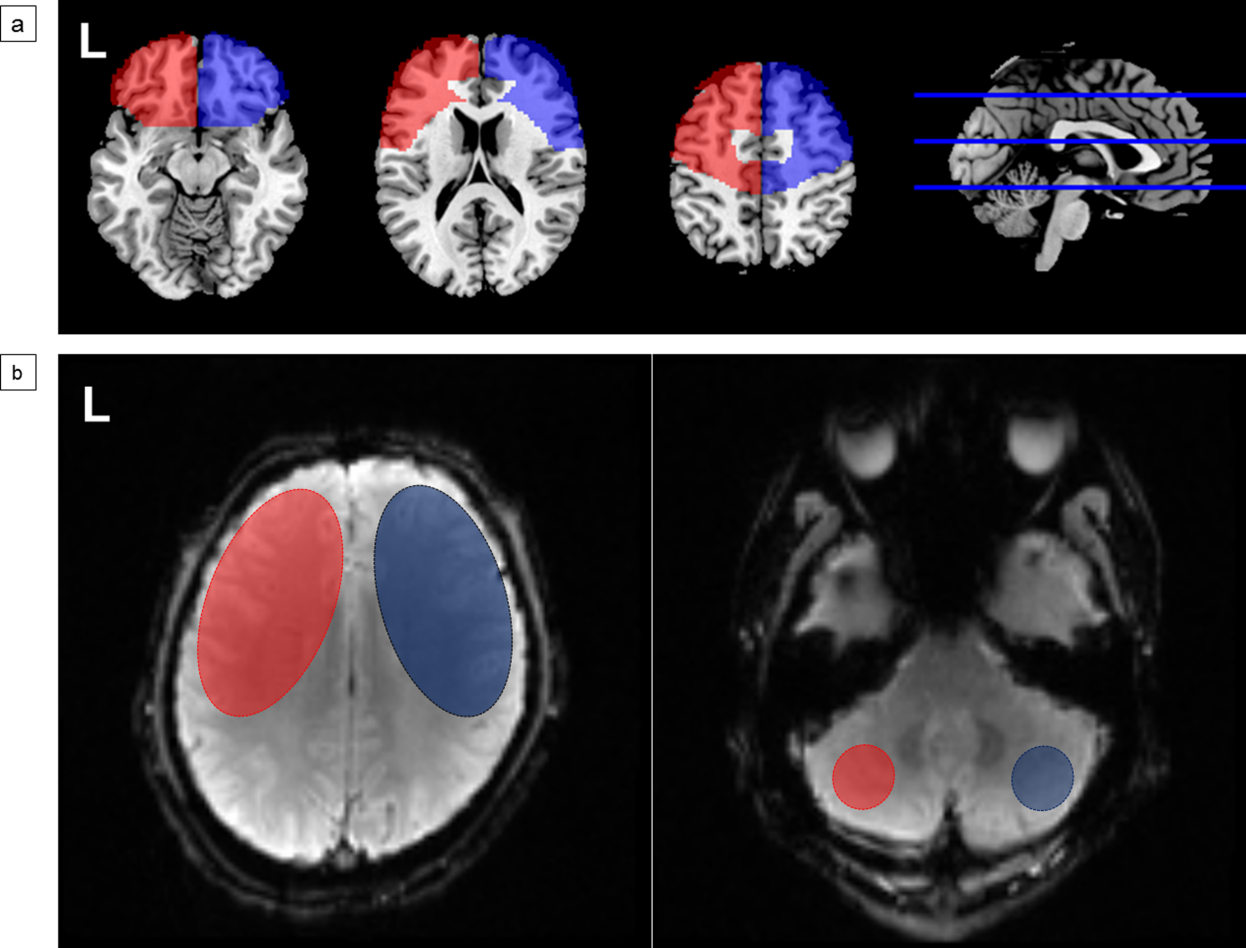
**Schematic images of (a) the frontal mask extraction of resting-state-functional magnetic resonance imaging data and (b) the quantitative measurement of perfusion-weighted magnetic resonance imaging (PWI) data.** Regions of interest on PWI maps were drawn manually in the bilateral cerebral and cerebellar hemispheres on mean transit time (MTT), time-to-peak (TTP), cerebral blood volume (CBV) and cerebral blood flow (CBF) images respectively. (Red, left hemisphere; blue, right hemisphere).

The procedure for calculating ALFF was based on previous studies.[8,10] The filtered time series were transformed to the frequency domain using fast Fourier transform (FFT) (parameters: taper percent = 0, FFT length = shortest). Since the power of a given frequency was proportional to the square of the amplitude of this frequency component, the square root was calculated by the power spectrum and averaged across 0.01–0.08 Hz at each voxel. For normalization, the ALFF of each voxel was divided by the global mean ALFF values for each subject. For comparison, the blood supply area of regions of interest (ROIs) of ALFF images should be consistent with those of PWI which were supplied by the middle cerebral artery (see below). In addition, in order to minimize the potential bias introduced by the posterior circulation, the bilateral frontal lobes were finally chosen as ROIs of ALFF images. The left and right frontal masks, which were extracted from WFU PickAtlas toolbox 3.0 (http://www.ansir.wfubmc.edu), were overlaid onto the global mean ALFF maps by using MRIcron software, respectively, and eventually the bilateral frontal lobe ALFF values of each patient were obtained (Fig 1A).

#### Perfusion-weighted MRI data processing

For quantitative analysis, the regions of interest (ROIs) in the cerebral hemispheres (mean size: 1500 mm^2^) were drawn manually on the first section above the top slice of the bilateral lateral ventricles. The ROIs in the cerebellum (mean size: 200 mm^2^) were aimed at minimizing the variances in arterial input function related to the bolus delay among the patients.[20] To analyze ROIs of PWI, the data were downloaded to workstation (Siemens Syngo Via20) for image postprocessing by using MR Neuro Perfusion software (Siemens, Erlangen, Germany). The values of CBV and CBF were calculated by dividing by cerebellum value of same side and the values of MTT and TTP were calculated by subtracting cerebellum value of same side. For example, detecting CBV value of right hemisphere ((CBV value of right frontal lobe/CBV value of right cerebellar hemisphere) × 100%) and detecting MTT value of right hemisphere (MTT value of right frontal lobe—MTT value of right cerebellar hemisphere) (Fig 1B).

### Statistical analysis

The independent-samples *t*-test was used to compare the frontal lobe ALFF between MMD patients and NC. The paired *t-*test was used to compare pre- and postoperative frontal lobe ALFF, MTT and TTP among MMD patients. The correlations between frontal lobe ALFF and PWI parameters (CBV, CBF, MTT and TTP) were assessed using Pearson’s correlation coefficient. All analyses were evaluated with a significance level of 0.05.

## Results

The basic information of patients (age, sex, scanning intervals, clinical presentation and Suzuki stage) and surgical interventions are summarized in the Table 1.

**Table 1:** Characteristics of patients (n = 30).

Patient	Age, y	Sex	Scanning Intervals^a^, m	Clinical Presentation	Suzuki Stage^b^		Surgical Interventions
No.					Left	Right	
1	25	M	4	TIAs	II	I	Left EDAS
2	27	M	4	SAH	III	IV	Left STA-MCA
3	59	M	3	TIAs	IV	II	Left EDAS
4	25	M	4	TIAs	IV	II	Left EDAS
5	26	F	5	IVH	III	V	Left STA-MCA
6	32	F	3	TIAs	III	II	Left EDAS
7	28	F	3	Stroke	V	V	Left EDAS
8	44	M	4	TIAs	0	VI	Right EDAS
9	32	M	6	TIAs	III	III	Left EDAS
10	22	M	3	Stroke	VI	VI	Left EDAS
11	42	F	3	IVH	II	II	Right STA-MCA
12	39	F	4	TIAs	III	IV	Left EDAS
13	41	M	4	Stroke	IV	III	Left STA-MCA
14	33	M	4	Stroke	II	II	Left EDAS
15	43	F	4	Stroke	III	III	Left EDAS
16	39	M	3	TIAs	III	III	Left EDAS
17	19	F	4	TIAs	IV	IV	Left EDAS
18	44	F	4	Stroke	III	IV	Left EDAS
19	41	F	3	Stroke	V	IV	Left EDAS
20	26	F	4	TIAs	IV	IV	Right EDAS
21	23	M	4	TIAs	IV	IV	Right STA-MCA
22	33	F	5	TIAs	III	VI	Left STA-MCA
23	46	M	6	TIAs	IV	II	Left STA-MCA
24	29	F	5	Stroke	V	V	Left EDAS
25	34	M	3	TIAs	IV	III	Left EDAS
26	34	M	3	Stroke	IV	V	Left STA-MCA
27	51	F	4	TIAs	IV	II	Left EDAS
28	45	F	3	TIAs	IV	II	Right EDAS
29	44	F	3	IVH	IV	IV	Left EDAS
30	43	F	4	Stroke	IV	II	Right EDAS

^**a**^ Scanning intervals: scanning time between pre- and post-operative rs-fMRI and PWI imaging

^**b**^ Suzuki stage[26]: Suzuki and Takaku first divided the angiographic features of moyamoya disease into six stages, reflecting the severity of the disease.

Abbreviation: F, female; M, male; TIAs, transient ischemic attacks; SAH, subarachnoid hemorrhage; IVH, intraventricular hemorrhage; EDAS, encephalo-dural–arterial synangiosis; STA-MCA, superficial temporal artery to middle cerebral artery.

### Different frontal lobe ALFF between MMD patients and NC group

**Fig 2:**
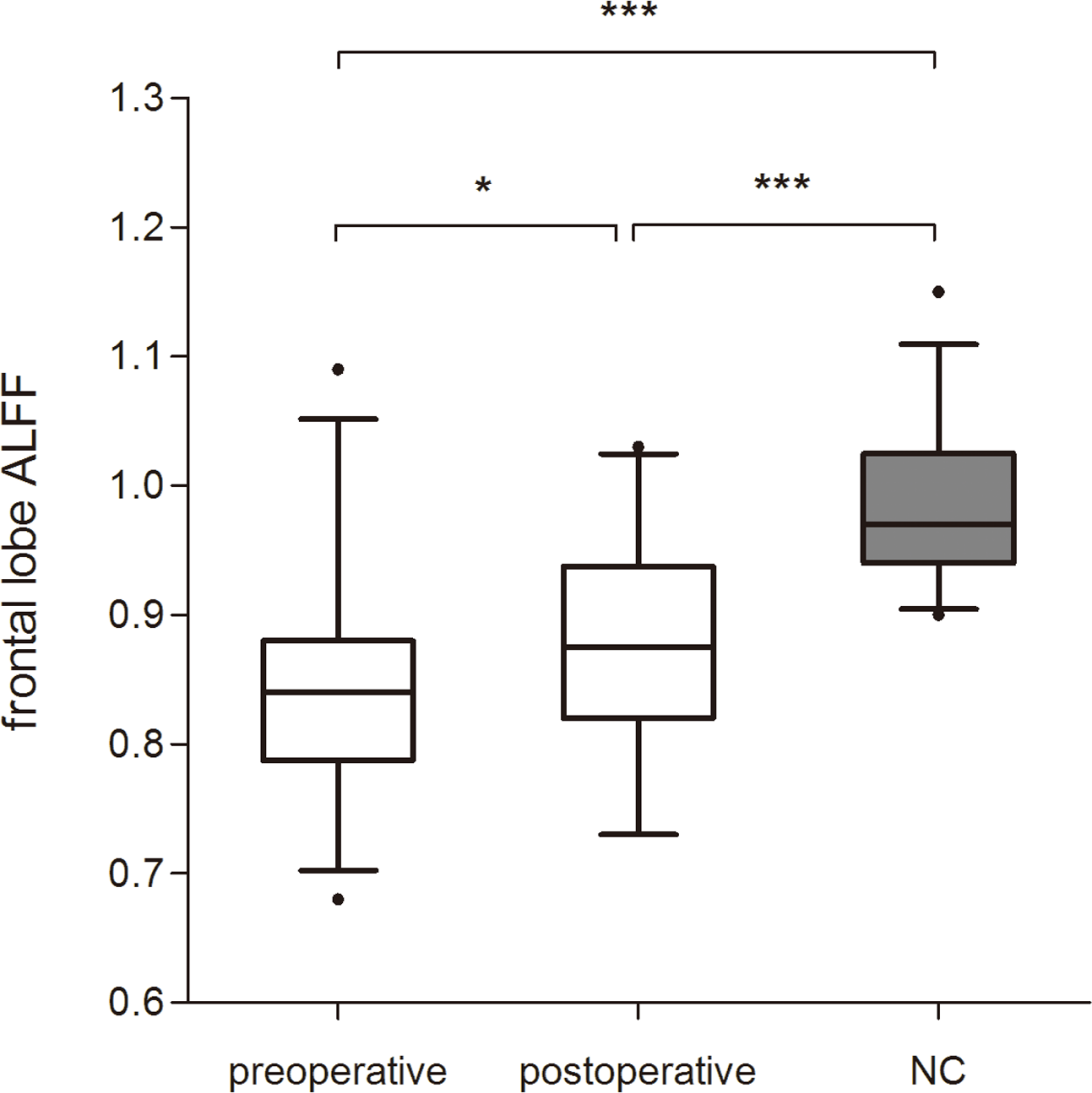
Comparison frontal lobe amplitude of low frequency fluctuations (ALFF) between normal controls (NC) and patients with MMD. The values of frontal lobe ALFF in NC were significantly higher than those in patients before and after revascularization surgery (independent-samples t-test, p < 0.001; p < 0.001, respectively). Postoperative values of frontal lobe ALFF show moderate improvement compared with preoperative values in patients (paired t-test, p = 0.011). The box-plot shows the median (line in box) and interquartile range between NC and patients, pre- and postoperatively. *p < 0.05, ***p < 0.001.

Pre- and postoperative frontal lobe ALFF in MMD patients were 0.84 ± 0.08 (standard deviation) and 0.88 ± 0.08, respectively; postoperative frontal lobe ALFF showed moderate improvement (paired t-test, p = 0.011; Fig 2). Frontal lobe ALFF in the NC group (0.98 ± 0.05) were significantly higher than that in MMD patients before (independent-samples t-test, p < 0.001; Fig 2) and after (independent-samples t-test, p < 0.001; Fig 2) revascularization surgery.

### Correlations between frontal lobe ALFF and PWI parameters

**Fig 3:**
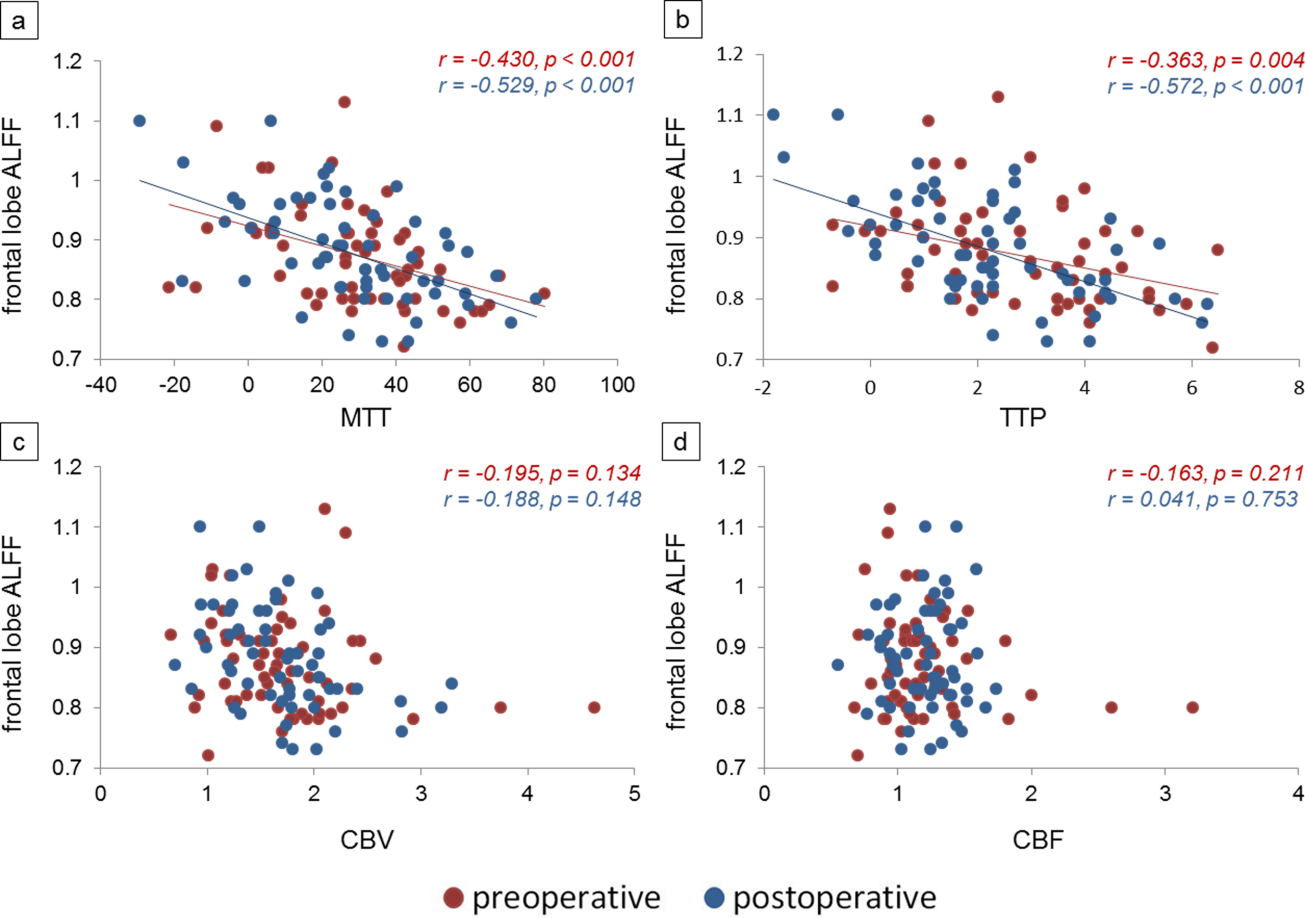
**Scatter plots of correlations between frontal lobe amplitude of low frequency fluctuations (ALFF) and (a) mean transit time (MTT), (b) time-to-peak (TTP), (c) cerebral blood volume (CBV) and (d) cerebral blood flow (CBF) in patients with MMD.** Preoperative (red) and postoperative (blue) values displayed under the same coordinate.

**Fig 4:**
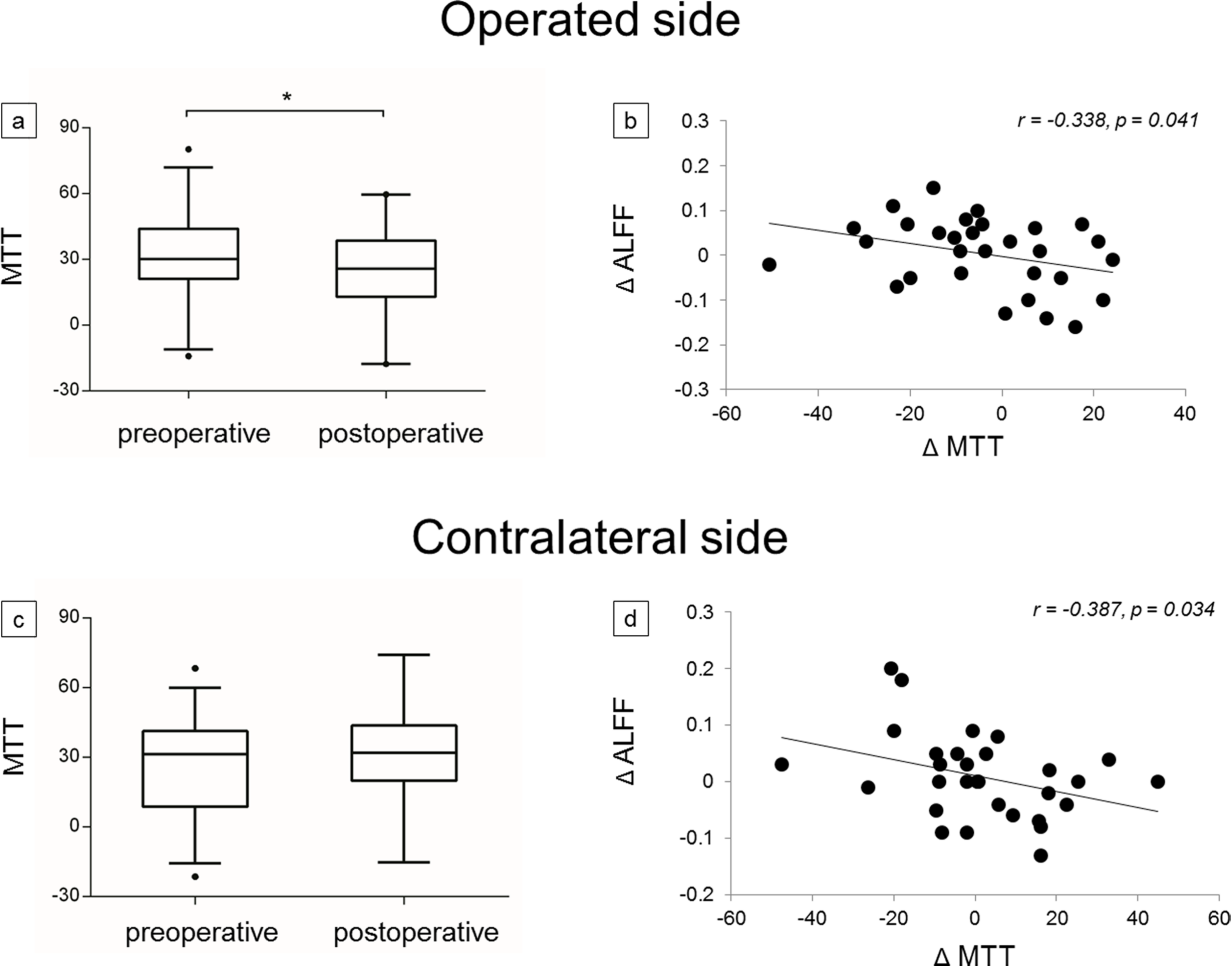
**Comparison between pre- and postoperative mean transit time (MTT) on both (a) operated side and (c) contralateral side. Scatter plots of correlations between Δ amplitude of low frequency fluctuations (ΔALFF) and ΔMTT on both (b) operated side and (d) contralateral side.** The box-plot shows the median (line in box) and interquartile range between pre- and postoperative MTT, Δ = postoperative values minus preoperative values, *p < 0.05.

**Fig 5:**
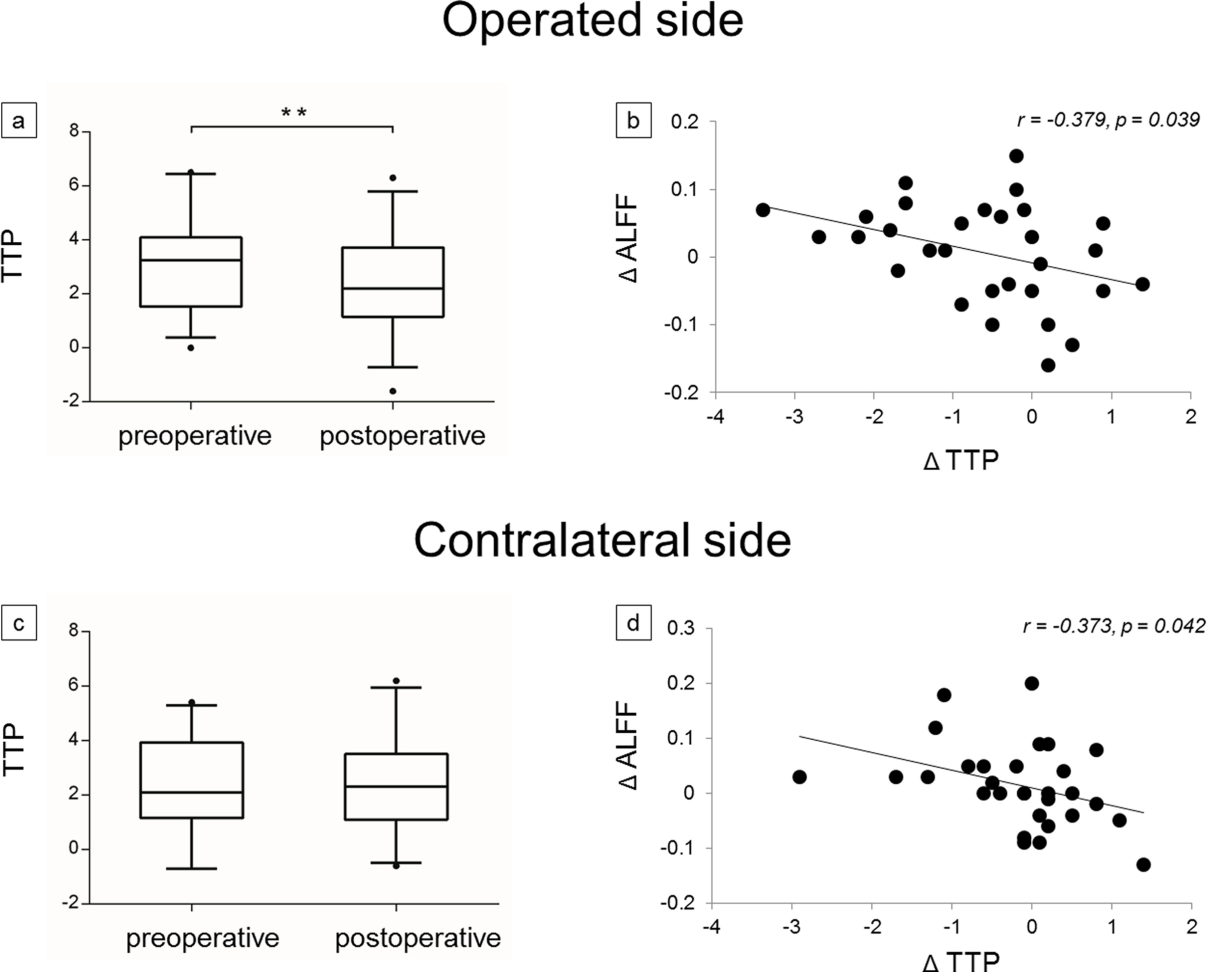
**Comparison between pre- and postoperative time-to-peak (TTP) on both (a) operated side and (c) contralateral side. Scatter plots of correlations between Δ amplitude of low frequency fluctuations (ΔALFF) and ΔTTP on both (b) operated side and (d) contralateral side.** The box-plot shows the median (line in box) and interquartile range between pre- and postoperative TTP, Δ = postoperative values minus preoperative values, **p < 0.01.

Some negative correlations were found between preoperative frontal lobe ALFF and MTT (r = -0.430, p < 0.001; Fig 3A), between preoperative frontal lobe ALFF and TTP (r = -0.363, p = 0.004; Fig 3B), between postoperative frontal lobe ALFF and MTT, (r = -0.529, p < 0.001; Fig 3A) and between postoperative frontal lobe ALFF and TTP (r = -0.572, p < 0.001; Fig 3B). Pre- and postoperative frontal lobe ALFF had no correlations with CBV (p = 0.134; p = 0.148, respectively) and CBF (p = 0.211; p = 0.753, respectively). (Fig 3C and 3D). The frontal lobe ALFF showed significant correlations with MTT and TTP rather than with CBV and CBF; thus, we focused on MTT and TTP for further investigation. We attempted to determine whether changes in frontal lobe ALFF corresponded with those in MTT and TTP after revascularization surgery. The postoperative values of MTT and TTP on the operated side were significantly shortened (p = 0.024; p = 0.002, respectively; Figs 4A and 5A), but no significant changes of those on the contralateral side were observed (p = 0.375; p = 0.350, respectively; Figs 4C and 5C). Nevertheless, negative correlations were both found between frontal lobe ΔALFF and ΔMTT on both operated side (r = -0.338, p = 0.041; Fig 4B) and contralateral side (r = -0.387, p = 0.034; Fig 4D) and between frontal lobe ΔALFF and ΔTTP on both operated side (r = -0.379, p = 0.039; Fig 5B) and contralateral side (r = -0.373, p = 0.042; Fig 5D). (Δ = postoperative values minus preoperative values).

## Discussion

**Fig 6:**
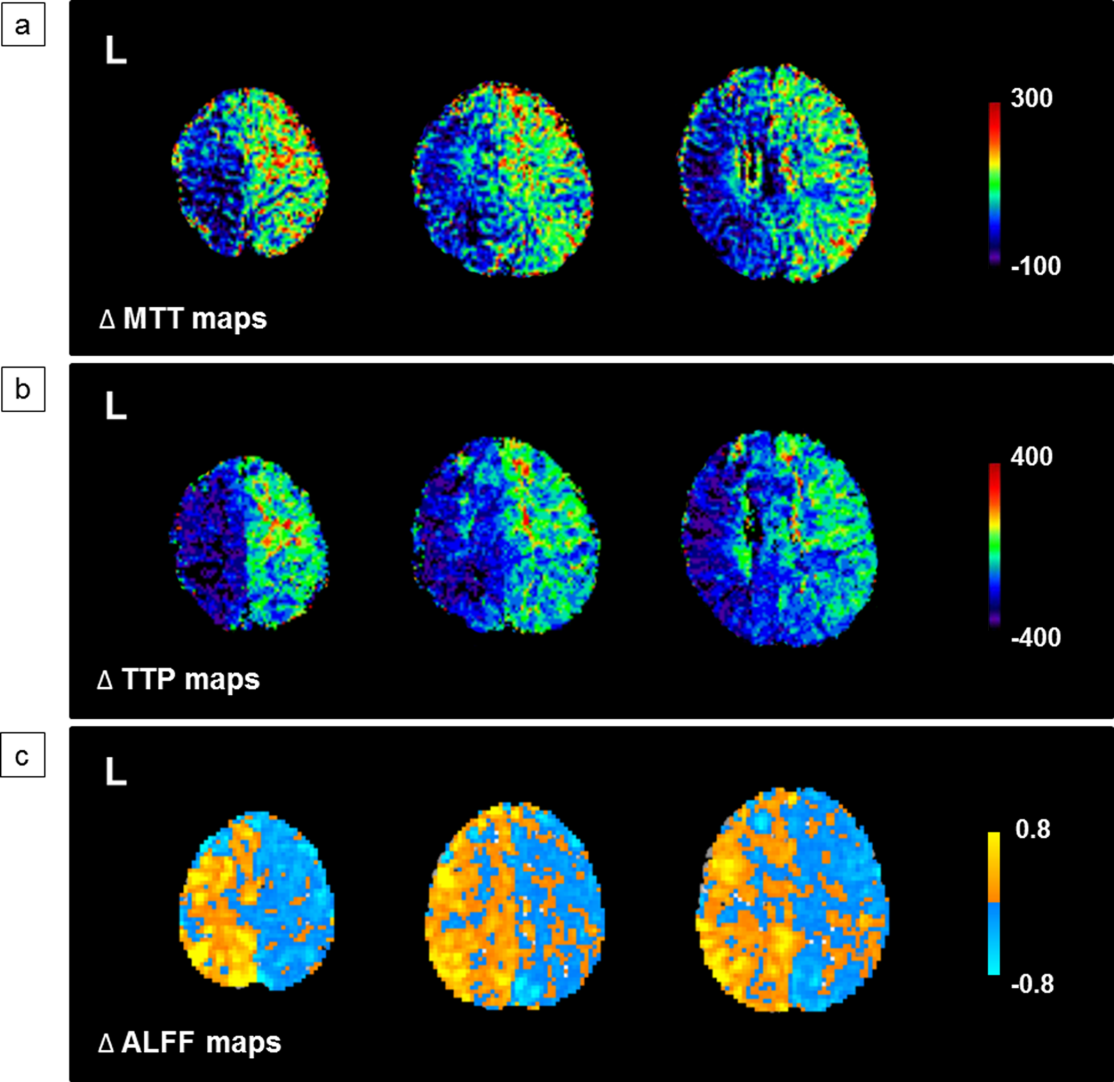
A representative case is shown. A 25-year-old man with moyamoya disease had undergone left revascularization surgery 5 months earlier. (a) Δ mean transit time (ΔMTT) maps, (b) Δ time-to-peak (ΔTTP) maps and (c) frontal lobe Δ amplitude of low frequency fluctuations (ΔALFF) maps were obtained. ΔMTT maps and ΔTTP maps show a significant shortening in the left hemisphere after revascularization surgery (cold color) and frontal lobe ΔALFF maps show a prominent augmentation in the same hemisphere (warm color). Δ = postoperative images minus preoperative images.

In this study, we compared the values of frontal lobe ALFF between MMD patients and a NC group and further investigated the correlation between the frontal lobe ALFF and PWI parameters before and after revascularization surgery in the patients. Frontal lobe ALFF in MMD patients were significantly lower than those in the NC group. Although frontal lobe ALFF in patients improved moderately after revascularization surgery, these postoperative values still remained prominent lower than those of the NC group. Additionally, the reduced values of frontal lobe ALFF in patients correlated well with the prolonged MTT and TTP and varied with the altered values of MTT and TTP after revascularization surgery (Fig 6). These findings support the hypothesis that ALFF could be affected by the abnormal cerebral perfusion in cerebrovascular diseases.

Prior neuroimaging studies have shown that low-frequency BOLD signal fluctuations mainly derived from spontaneous neural activity.[27,28] However, some physiological factors, such as CBF, could influence BOLD signals.[29,30] The magnitude of BOLD responses increases with incremental global CBF during task performance[31,32] and BOLD signals also correlate with CBF during rest in normal controls.[33] We speculate that the decreased baseline CBF in patients with MMD may induce the decreased resting-state BOLD responses. Moreover, the reduced ALFF may have some relation to the impaired CVR in patients with MMD. CVR, defined as the increase in CBF in response to a given vasodilatory stimulus (breath-holding or hypercapnic changes in end-tidal CO_2_), is considered to reflect the ability of the cerebral vessels to increase CBF upon reduction of cerebral perfusion pressure.[34] As the disease progresses, more blood flows towards areas of the brain capable of decreasing vascular resistance in response to a vasodilatory stimulus (the steal phenomenon), which could in turn cause a decrease in BOLD signals in the areas of the brain where vascular resistance was already minimized.[35,36,37,38,39] Compared with the NC group, the CVR in the bilateral hemispheres of patients were impaired to varying degrees, which may have reduced the resting-state BOLD responses. Therefore, we speculate that the reduced frontal lobe ALFF may be related to the abnormal cerebral perfusion in patients with MMD. Our findings showed that frontal lobe ALFF were moderately improved after revascularization surgery. However, these postoperative values in patients still remained markedly lower than those of the NC group. Noguchi and colleagues[21] reported that revascularization surgery had no significant effect on differences in arterial spin labeling values in patients with MMD. They speculated that revascularization surgery might not provide sufficient blood flow that was comparable to that in healthy subjects.

In this study, we found good correlations between frontal lobe ALFF and MTT and TTP before and after revascularization surgery. Eicker and colleagues[40] found that patients with impaired CVR had prolonged MTT and TTP in patients with internal carotid artery (ICA) occlusion. Other studies showed that the increased MTT correlated well with the decreased CVR in patients with occlusive cerebrovascular disease[41,42]. According to these studies, MMT and TTP should be predictive parameters of PWI for assessing reduced CVR, which have been evaluated based on the principle of BOLD signal fluctuations in patients with MMD[35,36,37,38,39]. In the present study, there was no correlation between frontal lobe ALFF and CBV or CBF. It has been showed that some resting-state BOLD signal fluctuations could be due to vasomotor oscillations in normal subjects.[43] In MMD, the impaired vasoconstriction or vasodilatation capacity may cause abnormal CVR. It has been reported that the reduced CVR correlated well with MTT, but not with CBF and CBV obtained by perfusion MR imaging[44,45] or by perfusion computed tomography[41] in patients with symptomatic ICA occlusion. They speculated that neither CBF nor CBV would alter linearly along with the reduced capillary perfusion pressure. In the early stage of MMD, an elevation of CBV and a prolongation of MTT occur in the brain tissue distal to the stenosis or occlusion to maintain CBF due to the mechanism of autoregulatory vasodilatation. With further reduction of capillary perfusion pressure, vasodilatation becomes maximal and CBF then begins to decrease. It thus appeared that CBF and CBV do not change linearly with the reduced capillary perfusion pressure. Furthermore, we found that no matter how the postoperative values of MTT and TTP varied (either shortened or prolonged), significant negative correlations between changes in PWI parameters and changes in ALFF were still observed respectively on both operated side and contralateral side after the unilateral procedures. Thus, according to our results, the sources of resting-state BOLD signal fluctuations may not only be neural activity, but also baseline cerebral perfusion status.

Notably, rs-fMRI has been used to investigate cerebral perfusion of cerebrovascular disease, such as acute stroke and MMD. Lv and colleagues[4] found a good correlation between MTT and the temporal-delayed resting-state BOLD signals in patients with acute stroke, by using time-lagged correlations. Similarly, Christen and colleagues[5] found that TTP was closely related to the temporal-delayed resting-state BOLD signals in patients with MMD. Jahanian and colleagues[46] found a strong linear correlation between data extracted from rs-fMRI and the BOLD percentage signal change during breath-holding challenge. It has been showed that the flow of systemic low-frequency oscillations derived from rs-fMRI did to a large extent represent the blood flow measured with PWI in normal controls.[47] Moreover, a recent study was reported that abnormal CVR had significant impacts on task-related BOLD fMRI activations in patients with MMD.[48] Based on the above studies, it is possible that drawing conclusions in the absence of considering the altered cerebral perfusion status could lead to misinterpretation of rs-fMRI results in steno-occlusive cerebrovascular diseases.

There were several limitations to our study. Firstly, it was challenging to draw the middle cerebral artery territories of the frontal lobes accurately on PWI maps; therefore, we selected relatively large ROIs on the first section above the top slice of the bilateral lateral ventricles. However, potential biases may have been introduced by including parts of the occipital lobes, which were supplied by the posterior circulation. Secondly, it remains possible that some ischemic or hemorrhagic lesions were not detected in FLAIR examinations. True values of PWI parameters and frontal lobe ALFF may therefore have been distorted. Thirdly, it was challenging to evaluate the true arterial input function in patients with MMD. Thus, we used cerebellar values as reference,[20] because the cerebellum was supplied by the posterior circulation and was not markedly influenced by revascularization surgery.[49] Finally, some self-reports of the patients’ in-scanner state were not available during the rs-fMRI scanning, which may have introduced some biases in this retrospective study.

## Conclusions

The present study demonstrates that chronic cerebral hypoperfusion status has great effect on the ALFF revealed by rs-fMRI in patients with MMD. Further investigations are needed to better understand the hemodynamic changes in rs-fMRI studies of steno-occlusive cerebrovascular diseases.
